# When is right time? Reassessing elective sigmoid resection in recurrent diverticulitis

**DOI:** 10.1097/MS9.0000000000004425

**Published:** 2025-12-03

**Authors:** Momina Inayat, Aleeza Khalid, Muddassir Khalid

**Affiliations:** Department of Medicine, Nishtar Medical University, Multan, Pakistan

*Dear Editor,*

Globally, recurrent diverticulitis still poses a problem for clinical judgment and healthcare systems. The management of diverticular disease has grown in importance as a public health concern due to the aging population and its increasing prevalence. Recurrence occurs in about 25% of patients with diverticular disease, and it is more common in people over 60 years of age (50%)^[1]^. In Germany, hospitalizations for diverticular disease topped 130 000 cases annually, and in the US, the annual cost is estimated to be USD 2.5 billion. These numbers highlight the worldwide burden of illness and the pressing need to improve the timing of elective sigmoid resection, a choice that has a direct bearing on morbidity, quality of life, and the distribution of healthcare resources.

In laparoscopic sigmoidectomy for diverticular disease, conversion rates and results are strongly influenced by the stage of the disease and the timing of the procedure. The best time to operate on patients with complex acute or chronic diverticulitis is when there is no inflammation. Due to a lack of randomized controlled evidence, European and American societies have differing recommendations regarding the best time to perform resection. According to a recent meta-analysis, delayed elective resection has lower conversion rates and shorter operating and hospitalization times than early surgery. Furthermore, when compared to traditional laparoscopy, recent developments like robotic colectomy have demonstrated promise in lowering conversion to open surgery and postoperative complications^[2]^.

Elective sigmoid resection has been shown to improve quality of life in patients with recurrent, complicated, or persistent symptomatic diverticulitis. While surgical risks must be balanced against benefits, evidence suggests comparable complication rates between initial conservative management and elective surgery. Health-economic analyses also support delayed surgery, with colectomy after the fourth rather than the second episode in patients aged 50 years or older, resulting in fewer deaths, fewer colostomies, and cost savings of over USD 1000 per patient^[3]^. In younger patients, delaying surgery until after the fourth episode leads to fewer colostomies and savings exceeding USD 5000. The LASER randomized clinical trial (2025) further demonstrated that, although elective resection reduces recurrence rates, it does not significantly improve long-term quality of life compared to conservative treatment. These findings highlight the importance of individualized surgical timing guided by symptom burden, recurrence pattern, and overall quality of life^[4]^. These results emphasize the significance of individualized surgical scheduling based on burden symptoms, recurrence patterns, and general quality of life.

According to recent surveys of European colorectal surgeons, 89% of them agree that surgery is necessary for immunosuppressed patients, regardless of the number of episodes, and 95% agree that surgery is necessary for persistent symptoms^[5]^. A delayed approach – typically 6–8 weeks post-inflammation – is recommended for complicated disease involving fistulae, stenosis, or abscesses, while early elective resection may be considered in uncomplicated recurrent cases with significant symptoms or impaired quality of life. To identify recurrence and avoid emergency surgery, close outpatient monitoring with imaging when necessary is crucial during this observation period. Crucially, routine endoscopy 4–6 weeks postattack is no longer universally advised, promoting individualized care pathways and minimizing needless interventions. In conclusion, rather than using set numerical thresholds, a personalized, evidence-informed framework should be used to determine the optimal timing for elective sigmoid resection. Decision-making and resource use will be enhanced by incorporating cost-effectiveness data, clinical risk profiles, and patient-reported outcomes. In order to improve surgical timing, future research should concentrate on creating prospective risk-stratification tools that integrate imaging predictors and biomarkers. A worldwide agreement on customized surgical indications is essential to minimize variability, improve quality of life, and save healthcare costs.

TITAN Guidelines: This manuscript is in compliant to the TITAN Guidelines, 2025, declaring no use of artificial intelligence^[6]^.

## Data Availability

Not applicable.

## References

[1] Comparato G Di Mario F. Recurrent diverticulitis. J Clin Gastroenterol 2008;42:1130–34. 18936650 10.1097/MCG.0b013e3181886ee4

[2] Murdock PMW Venero AC Heidel RE Hale BW Russ AJ. Laparoscopic versus robotic elective sigmoid resection for complicated diverticulitis. JSLS 2025;29:e2024.00079. 10.4293/JSLS.2024.00079 PMC11967722 40182832

[3] Hawkins AT Wise PE Chan T. Diverticulitis: an update from the age old paradigm. Curr Probl Surg 2020;57:100862. 33077029 10.1016/j.cpsurg.2020.100862 PMC7575828

[4] Santos A Mentula P Pinta T. Sigmoid resection vs conservative treatment after diverticulitis: prespecified 4-year analysis of the LASER randomized clinical trial. JAMA Surg 2025;160:615. 40202724 10.1001/jamasurg.2025.0572 PMC11983291

[5] Girardin T Martin D Lázaro-Fontanet E. Swiss consensus on the management of acute diverticulitis. BJS Open 2024;8:zrad165. 38377168 10.1093/bjsopen/zrad165 PMC10878436

[6] Agha R Mathew G Rashid R. Transparency in the reporting of Artificial INtelligence – the TITAN guideline. Premier J Sci 2025;10:100082.

